# Evaluating the compatibility of predictive coding as a model of linguistic predictability effects in reading

**DOI:** 10.3389/fcogn.2026.1768534

**Published:** 2026-09-16

**Authors:** Allyson Copeland, Brennan R. Payne

**Affiliations:** 1 Department of Psychology, University of Utah, Salt Lake City, UT, United States; 2 Department of Communication Sciences and Disorders, University of Utah, Salt Lake City, UT, United States; 3 Interdepartmental Program in Neuroscience, University of Utah, Salt Lake City, UT, United States; 4 Center on Aging, University of Utah, Salt Lake City, UT, United States

**Keywords:** linguistic processing, N400, prediction in reading, predictive coding, predictive processing

## Abstract

Emerging work suggests that the computational architecture of predictive coding (PC), widely studied in visual perception, may be an appropriate model for understanding predictability effects in real-time reading (i. e., the influence of a preceding sentence context on current word processing via putative anticipatory mechanisms). We evaluated four hypotheses derived from PC models focused on: (1) bottom-up prediction error, (2) top-down sensory prediction, (3) biological plausibility, and (4) hierarchical inference. We argue that many empirical effects of predictability during reading are generally consistent with PC model hypotheses, such as ERP N400 context effects and facilitated recognition and reading times, while others are challenging to reconcile, such as the frontal positivity ERP effect to plausible prediction violations and emerging work examining brain-behavior dynamics in natural reading. Overall, we suggest that while PC applied to linguistic processing has some important explanatory utility, it likely does not reflect the sole core mechanism of the language architecture.

## 1 Introduction

A major aim of psycholinguistics is understanding how contextual information (i.e., any information preceding the current input) influences specific aspects of word and sentence processing (e.g., Kutas and Hillyard, 1980). Predictive processing offers a parsimonious mechanism for rapid linguistic processing–an efficient way to keep up with the rapidly unfolding, oft-ambiguous and noisy input of real-world language comprehension (e.g., Kuperberg and Jaeger, 2016; Wong et al., 2025). As such, prediction has been the focus of much research in language comprehension, including work from reading and speech studies using electrophysiology, eye-tracking, and behavioral measures (e.g., Kuperberg and Jaeger, 2016; Pickering and Gambi, 2018; Clark, 2024; Huettig, 2025). This research consistently shows that predictable words are easier to recognize, influence eye movement control (e.g., shorter fixation durations, increased word skipping), and show facilitated neural processing (for a recent review, see Wong et al., 2025). Nevertheless, while there has been some degree of convergence across experiments and methodologies in the facilitative nature of prior context, it remains debated whether prediction is a ubiquitous feature of sentence processing. In part because of conceptual divisions, different methodologies often yield conflicting conclusions regarding the necessity, time-course, and granular details of predictive processing.

To overcome such empirical and theoretical divergences, computational modeling has been at the forefront of theoretical development in linguistic processing. Indeed, there is a robust and widespread literature attesting to the theoretical utility of computational modeling approaches in reading research at large (e.g., E-Z Reader model, Reichle, 2011; Simple-Recurrent Neural Networks, Elman, 1990, 1991; for a recent overview, see Reichle, 2021). Emerging work suggests that predictive coding (PC) architecture may be an appropriate model for understanding predictability effects in language processing (e.g., Huettig, 2015). However, formal computational implementations of predictive coding have received only limited attention and application to online linguistic processing until recently (e.g., Ohams et al., 2026; Nour Eddine et al., 2024). PC models center *top-down expectations* as the primary currency of perception (e.g., Huang and Rao, 2011). Instead of bottom-up sensory information being carried up to higher levels of the cortical hierarchy, it is the residual (unpredicted) information that gets propagated up (Heeger, 2017). While there have been recent attempts to characterize the empirical nature of predictability effects (see Wong et al., 2025), there has yet to be a systematic evaluation of whether PC models can sufficiently account for empirical evidence of predictability effects in reading. That is the primary goal of this conceptual analysis. In what follows, the remainder of Section 1 frames two of the most prominent theoretical questions in psycholinguistics on the locus and time-course of predictability effects. Section 2 then provides a brief overview of extant predictive coding models. Finally, Section 3 evaluates whether PC can fruitfully account for current empirical evidence surrounding these questions.

### 1.1 Outstanding theoretical questions in predictive language processing

Following a long and historically vital debate about the very existence of anticipatory mechanisms in language processing (for relevant discussions see Kutas et al., 2014; Kuperberg and Jaeger, 2016), the field has now largely moved beyond asking *whether* we predict. Instead, modern psycholinguistics focuses on understanding mechanistic questions of *when, why*, and *how* we predict (Huettig, 2015). For instance, Van Petten and Luka (2012) suggest focusing on delineating the form, nature, and mechanisms of prediction. Addressing such questions requires navigating a fragmented theoretical landscape as a breadth of different (and only partially overlapping) terms are often evoked by the concept of “prediction” in the literature—e.g., expectation, anticipation, prospection, preparation, foresight, and context effects [list reproduced from Huettig (2025), p. 13]. To bring clarity to this landscape, it is critical to differentiate predictability as a stimulus feature from prediction as a cognitive process enacted by the reader (DeLong et al., 2014b). Under this framework, prediction refers to cognitive mechanisms that prepare and support the processor for likely upcoming input *in the future* (DeLong et al., 2014b). In contrast, predictability is often considered an inherent structural feature of the *stimulus* itself—i.e., as how likely it is that a given word *can be* predicted from preceding context (e.g., Wong et al., 2025; DeLong et al., 2014b). Predictability is often operationalized as cloze probability (Taylor, 1953; for recent overviews see Wong et al., 2025; Staub, 2015)^1^ though recent work has operationalized stimulus predictability via surprisal estimates derived from large language models (LLMs; e.g., Brothers and Kuperberg, 2021; Michaelov et al., 2024). Regardless of how these stimulus features are operationalized, evaluating how and when the language processing system leverages this information requires addressing two deeply intertwined theoretical debates: the precise *locus* of these effects within the language processing stream and the extent to which such predictive processing mechanisms are an *obligatory* feature of language comprehension.

#### 1.1.1 What stage(s) of linguistic processing do predictability effects reflect?

Many models of word recognition in sentence processing posit serially-staged access, such that there is an explicit order to extracting information in real time (for a review see Traxler, 2011, Chapter 4). For example, in E-Z Reader, a model of eye movement control in reading, saccade programming (i.e., the signal to move the eyes) is generated by completion of an early stage of lexical processing (“familiarity check,” Reichle, 2021). Predictability (as a “lexical” stimulus feature) influences time to completion of this “familiarity check” stage and the second stage of lexical processing (full word identification). Only following lexical processing, can influences of message-level semantic and syntactic factors (e.g., plausibility, syntactic complexity) be observed. There's ongoing debate about whether and where predictability effects fall in this process timeline (for reviews see Staub, 2015; Wong et al., 2025). For instance, predictability effects can be difficult to localize on a given target word since they may emerge before foveal target fixation (Copeland and Payne, 2025) and persist into downstream processing (see for example discussion of Luke and Christianson (2016) in Section 3.2.2 below). More broadly, we can consider whether discrete staged views of processing are the best characterization and conceptualization of predictive processing. Alternatives have long existed to staged-access models, such as the class of Constraint-Based connectionist models (e.g., MacDonald et al., 1994) which allow for parallel activation and interaction across all representational information sources in sentence processing (e.g., lexical, semantic, pragmatic).

#### 1.1.2 Is prediction obligatory?

There have been varied perspectives on the role and importance of prediction for language processing. The more traditional conception is *lexical* prediction, or predicting a specific lexical candidate or linguistic item, in this case a particular word (for a recent review see Wong et al., 2025). This is typically formulated as an active, all-or-nothing kind of process, where a reader either activates a word before perceiving it or not (see also Kutas et al., 2011; DeLong et al., 2014a). A variation of this is (graded) pre-activation, where the reader passively activates partial information about likely upcoming input (for example, semantic or orthographic features; Brothers and Kuperberg, 2021; Federmeier, 2022; Luke and Christianson, 2016; Staub, 2015; Staub et al., 2015; Kutas and Federmeier, 2000). This kind of activation is rapid, mostly automatic, graded, and relies on *time* to spread across conceptual associations in long-term memory (Federmeier, 2022). This can be considered as a “landscape of activation,” where a diverse set of information becomes available, for instance across visual areas, the semantic memory system, and more (Federmeier, 2022, p. 16). It is typically framed as working in concert with more *active* prediction mechanisms, which may also include direct lexical pre-activation. In summary, some views hold prediction as a core feature (e.g., predictive coding, discussed in more detail below), while in other views it is seen as a “peripheral” feature helpful only in particular circumstances (Luke and Christianson, 2016, p. 23; Huettig, 2015). Summarized, open questions include (but are not limited to): (1) Is prediction a necessity in linguistic processing, or is it flexible and task-dependent? (2) Are linguistic predictions specific enough to be informative? We return to these questions in Section 3. First, in Section 2, we provide a brief introduction and overview of predictive coding frameworks.

## 2 Predictive coding: A dance between deviant and expected information

Information theory (Shannon, 1948) showed that communication can be made more efficient by exploiting redundancy. A familiar example is video compression, where instead of sending full pixel data in every frame, only changes across successive frames are typically encoded. In the same way, neural PC frameworks are centered on reducing redundancy by prioritizing “deviant” information. PC postulates it is the difference between the top-down expected signal (predictions) and bottom-up signal— *prediction error*—that gets transmitted along the cortical hierarchy. Therefore, PC is a specific proposal about the nature of cortical function and information processing. Length constraints preclude a detailed review of PC model architecture (for reviews see Millidge et al., 2021; Sprevak, 2024). Below, we provide a brief sketch of the PC model and important model features (see Figure 1).

**Figure 1:**
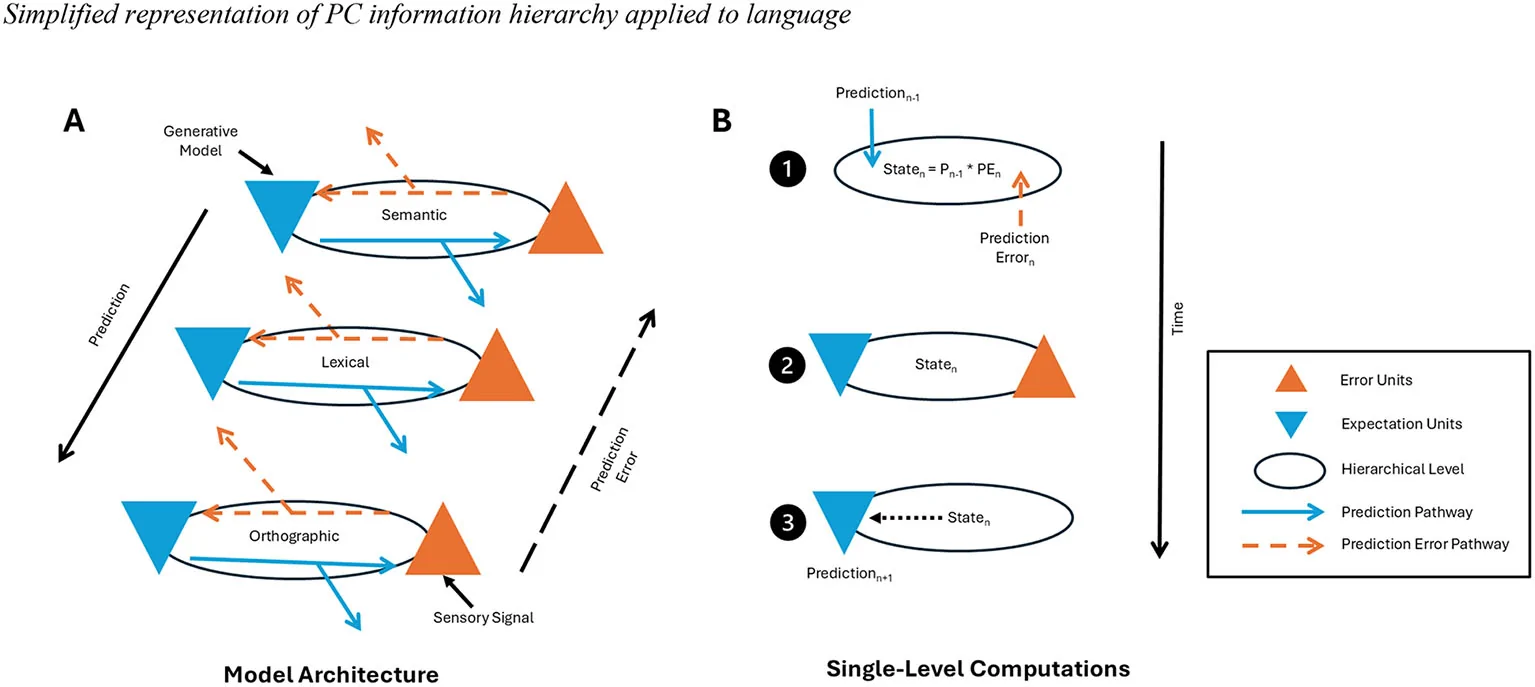
**(A)** Model architecture. Panel A represents a simplified view of the spatial organization of model parameters in a hierarchical predictive coding architecture. Each hierarchical level (orthographic, lexical, semantic) contains expectation/prediction units and prediction error units. Expectation units represent prediction information inferred from the superordinate level (or generated from the internal model at the highest level). Error units encode residual information that was not explained away that gets sent up the hierarchy to superordinate levels. Dashed (orange) arrows represent the flow of prediction error, such that expectations modify the error that is propagated up the hierarchy. Solid (blue) arrows represent the flow of predictions, such that each level's error modifies the expectations that are sent down the hierarchy to subordinate levels. This figure was adapted from Huettig (2025), Nour Eddine et al. (2022), and Walsh et al. (2020). **(B)** Single-level computations. Panel B represents the temporal ordering of computations that occur within a single level of the hierarchy across one iteration of the model. The precise method of computations for these updates depend on the implementation (which we are not committing to here). The terminology used for model parameters is not consistent across the literatures. State units are conceptually identical to expectation units or prediction units. In order to simplify the model here, “state” as represented in Panel B is only marked separately as a way to visually represent that it is not the state reproduced exactly that gets transmitted down the hierarchy (but the combination of state activity and the previous level's predictions, called top-down bias). In this figure, *n* refers to the current iteration. Step 1: State units (at one hierarchical level) are first updated based on the predictions from the previous iteration and prediction error from the subordinate level. Step 2: Residual information *then* gets computed to be sent up the hierarchy (prediction error) and retained at the current level for use at the next iteration (top-down bias). Step 3: *Finally*, predictions (top-down reconstruction) are generated for use on the next iteration based on the current state and superordinate level's input.

Perception relies on being able to infer meaning (and causes) from effects (noisy, irregular sensory information). One solution toward handling this ambiguity in sensory information is to have perception itself as causal inference (with theoretical origins at least as early as von Hemholtz; Lupyan and Clark, 2015; Millidge et al., 2021). Prediction error minimization is a tractable method for dealing with this uncertainty. By utilizing generative models that encode an internal representation of the environment, the brain constantly compares its predictions against incoming sensations to minimize the discrepancy between its internal model and the “actual external world” (Hohwy, 2013; see Figure 1B). In this view, the world itself “supervises” this inferential process by serving as *feedback* on internal generative models (i.e., priors; Hohwy, 2013).^2^

Linking to Marr's (1982) levels of analysis, prediction error minimization serves as a method toward achieving computational-level goals, here to explain perception via real-time inference about latent states (Millidge et al., 2021). The algorithmic level is represented by hierarchically organized layers of prediction and error units (see Figure 1A). In PC models, processing is largely implemented by feedback (backwards) connections (Figure 1, “Prediction Pathway”), which transmit predictions from higher to lower levels, while feedforward connections (Figure 1, “Prediction Error Pathway”) transmit prediction error upwards to higher levels (Lupyan and Clark, 2015; Millidge et al., 2021). Critically, this hierarchy can instantiate a wide variety of predictions at multiple scales (Millidge et al., 2021). Kersten et al. (2004) developed a useful heuristic for this hierarchical communication called “shut up and stop gossiping”:

High-level areas may explain away the sensory input and tell the lower levels to “shut up.” Alternatively, high-level areas might sharpen the responses of early areas by reducing activity that is inconsistent with the high-level interpretation; that is, high-level areas tell competing representations in lower areas to “stop gossiping.” (Friston, 2005 describing Kersten et al., 2004, p. 829)

Put another way, it is because of predictions that prediction errors are reduced (or minimized), and why expected inputs facilitate processing (smaller prediction errors).

The implementation level is represented via functionally distinct entities for these units (Millidge et al., 2021). These two classes of neurons are constituted by (1) those that encode predictions about sensory states (bottom-up input) called *state units* (or “the internal representation being inferred,” also called *expectation* or *prediction units*) and (2) those that encode information deviating from those predictions called *error units* (Walsh et al., 2020).

A key feature of PC models is that they can implicitly account for causal structure of the world via generative models and the hierarchical structure of levels. The cortical hierarchy includes both the previously discussed spatial dimension (i.e., levels) as well as a temporal dimension. Higher-level regularities (e.g., *invariant* abstract information) in the environment that unfold over longer timescales (e.g., orthographic regularities in English) are contained within information that moves down the hierarchy. In contrast, fast, dynamic regularities (e.g., first-person perspectival *variant* information) are contained within information that moves up the hierarchy (e.g., unfamiliar font). It is in these interactions between regularities at different time scales that the hierarchy reflects causal structure, therefore making *context-dependence* an integrated design feature (Hohwy, 2013). In other words, PC's spatiotemporal architecture necessarily accounts for the context of the current input during perceptual inference. To handle context-sensitive *uncertainty* (e.g., noisy/ambiguous input) PC models have a precision-weighting mechanism to estimate signal reliability by modulating prediction error weights. The system accomplishes this by evaluating how confident it is that a prediction error is genuine and worth minimizing, “assigning more or less weight to top-down expectations vs. bottom-up inputs” based on that evaluation (Lupyan and Clark, 2015, p. 281). Higher levels encoding more global, longer-term representations require higher precision error (large gain) to be updated compared to lower levels encoding more local, shorter-term representations, which are sensitive to relatively lower precision errors (Hohwy, 2013, p. 69). For example, in the classic audiovisual speech perception McGurk illusion (phoneme paired with an incongruent facial cue), your percept is fused across modalities (for a review see Alsius et al., 2018). Some models of Bayesian multisensory perception (e.g., Lindborg and Andersen, 2021) hypothesize that the resulting percept is influenced by precision weighting based on the reliability (e.g., signal-to-noise ratio) of each cue (i.e., auditory and visual). When there's lower prediction error gain (input is ambiguous), the system prioritizes prior expectations about how sounds are typically mapped to vocal production to aid in your inference. Therefore, the overall pattern of prediction error gain (increased weighting) or inhibition (decreased weighting) should mirror state-dependent noise or uncertainty (Hohwy, 2013).

Presently, precision-weighting is largely underspecified in its implementation, both in model and neurobiological terms. Given our conceptual assumptions about real-time dynamic modulation of predictions, we focus our interpretation of precision-weighting on bottom-up (i.e., stimulus-driven) real-time variability about sensory state uncertainty (Millidge et al., 2021; Bogacz, 2017). However, this is only one of several possible implementations—others include implicit learning of precisions in model parameters via training (Millidge et al., 2024), setting fixed expectations about variability (Millidge et al., 2024), and task-adaptive modulation or attention (Feldman and Friston, 2010). Longer-term updating of these weights serves other computational-level goals, such as learning (Rao and Ballard, 1999). We now turn to a critical discussion of how predictive coding has fared thus far as an explanatory mechanism of linguistic prediction in reading.

## 3 Does empirical evidence for predictability effects in reading support PC as a model of linguistic processing?

Language processing requires coordination across sensory and cognitive domains (e.g., memory, vision, hearing, attention) and levels of representation. Predictive coding offers one unified approach toward capturing these dynamics that have traditionally been modeled separately. This moves away from traditional “slices” of processing, which include unique models of word identification, sentence processing, discourse representation, and eye movement control (Reichle, 2015), toward complete, comprehensive models of reading, such as Über Reader (Reichle, 2021). As Reichle (2021) points out, “it is probably not possible to accurately simulate or understand any individual component of reading independently of the others” (Reichle, 2021, p. 443). Therefore, theoretical proposals aiming to be comprehensive, unified, and complete should receive earnest consideration. Predictive coding serves as a unified theory capable of explaining cognition across all domains, from early perception to social interactions. Specific to our purposes, predictive coding has the potential to bridge domains of processing and bridge empirical discrepancies across methods on the locus and time-course of lexico-semantic predictability effects.

Indeed, several researchers (and some philosophers) have recently advocated for, generated theoretical and implemented models for, and presented empirical evidence for PC as a model of linguistic processing (Lupyan and Clark, 2015; Bornkessel-Schlesewsky and Schlesewsky, 2019; Nour Eddine et al., 2022, 2024; Ryskin and Nieuwland, 2023; Kuperberg and Jaeger, 2016; Lewis and Bastiaansen, 2015; Silcox and Payne, 2025; Wang et al., 2023, 2025; Zhao et al., 2019; Fu and Gagl, 2025). For instance, recent attempts have been made to link predictive coding architecture to incrementally unfolding processing as reflected in word reading times (Ohams et al., 2026). The present analysis falls within a more general enterprise toward evaluating the neural and behavioral evidence for predictive coding (Millidge et al., 2021). For example, Walsh et al. (2020) comprehensively evaluated empirical support for PC models in visual perception. Here, we use this Walsh et al. (2020) framing to assess four primary hypotheses derived from PC models (hypotheses reproduced below exactly) within the language processing literature, primarily focusing on reading.^3^

1. Error-signaling neural responses to sensory stimuli should scale inversely with expectation.
2. Top-down signals represent sensory prediction.
3. At each level of the cortical hierarchy there are two functionally distinct neural subpopulations representing predictions and prediction errors.
4. Prediction error minimization is achieved through reciprocal exchange of error and prediction signals across levels—a process known as “hierarchical inference.”

This is intentionally a *selective* review meant to serve as a starting point for considering whether PC models capture predictability effects (others have recently covered predictability effects more exhaustively; see Wong et al., 2025; Staub, 2015), and as such will focus on plausible text-based stimuli and high temporal resolution methods capable of capturing temporal dynamics of prediction (i.e., a limited fMRI literature is included) with a specific focus on the reading literature. See Table 1 for a summary of empirical evidence evaluations within a PC framework organized by the four major hypotheses and with key references.

**Table 1:** Summary of major hypotheses, empirical evidence for predictability effects evaluated and consistency with predictive coding architecture.

Sub-hypotheses	Empirical evidence	Key references	Consistent?
**Hypothesis 1: Prediction Error** Error-signaling neural responses to sensory stimuli should scale inversely with expectation			
**Expectation suppression:** Reduced neural activity after presentation of expected stimuli	N400 graded by cloze probability	Kutas and Hillyard, 1980; Kutas et al., 2006	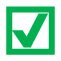
	Relationship with memory: Related information also activated by strong contexts (within-category N400 effects are smaller than between-category violations)	Federmeier and Kutas, 1999	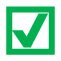
	Null orthographic neighborhood × constraint interaction	Payne et al., 2015	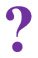
	Left anterior anticipatory negativity strongest to highly constraining contexts	León-Cabrera et al., 2024	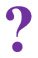
	RSVP: N400 effects at article preceding predictable targets	DeLong et al., 2005; Nieuwland et al., 2018; Rabovsky, 2020	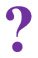
	Contextually graded N400 elicited at parafoveally viewing of targets	Stites et al., 2017; Copeland and Payne, 2025	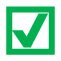
**Repetition suppression:** Neural activity should be reduced at repeated stimuli	Relationship with memory: Expected word activation reduced upon repetition and unpredicted words have short-lived representations	Rommers and Federmeier, 2018a,b	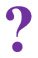
	Contextually graded parafoveal N400 is subsequently reduced upon foveation	Payne et al., 2019; Milligan et al., 2023	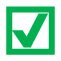
**Hypothesis 2: Prediction** Top-down signals represent sensory prediction			
	Behavioral indices of predictability benefits	Ehrlich and Rayner, 1981; Balota et al., 1985	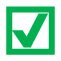
	Limited role of contextual constraint when processing unexpected stimuli	Luke and Christianson, 2016; Wong et al., 2024	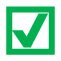
	Relationship with word form (preview benefits and orthographic neighbors)	Milligan and Schotter, 2025; Nour Eddine et al., 2024	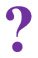
	**Omission responses:** Response to expected stimuli that were never actually presented; see Section 3.2.4 for more details	Staub et al., 2024	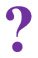
**Hypothesis 3: Biological Plausibility** At each level of the cortical hierarchy there are two functionally distinct neural subpopulations representing predictions and prediction errors			
	Left localization of predictability (MEG semantic context effects)	Wang et al., 2023, 2025	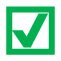
	Cortical information flow may be linked to oscillatory frequencies	Giraud and Arnal, 2018	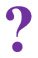
	Anticipatory alpha modulation	Jensen, 2024	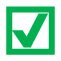
**Hypothesis 4: Hierarchical Inference** Prediction error minimization is achieved through reciprocal exchange of error and prediction signals across levels			
	Frontal positivity to unexpected targets	Lai et al., 2024	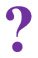
	Relationship with experience/expertise	Andrews et al., 2023; Federmeier et al., 2010	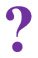
	Relationship with linguistic environment	Brothers et al., 2017; Wong et al., 2024	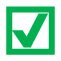

### 3.1 Hypothesis 1 (Prediction Error): Error-signaling neural responses to sensory stimuli should scale inversely with expectation

PC models propose that the information sent up the cortical hierarchy is prediction error (difference between current hierarchical level's state and top-down prediction). As such, the response signaling errors should decrease as prediction increases. Walsh et al. (2020) and others have identified three hypothesized effects that would indicate this dynamic: omission responses,^4^ expectation suppression, and repetition suppression.

#### 3.1.1 Expectation suppression

Expectation suppression is reduced neural activity after presentation of expected stimuli “on the basis of statistical regularities or prior cues” (Walsh et al., 2020, p. 244). In other words, predictability should reduce neural activity, since PC models explain away predicted signals (Huettig, 2025). In the language processing literature, expectation is operationalized primarily as cloze probability.

##### 3.1.1.1 Semantic access facilitation graded by predictability (N400 effects)

The most-widely studied language ERP is the N400, a centro-posteriorly distributed negativity that peaks ~400 ms after stimulus onset. The N400 is elicited by any meaningful (or potentially meaningful) stimulus such as words (lexical frequency: Rugg, 1990; concreteness: Barber et al., 2013; word position: Van Petten and Kutas, 1991; orthography: Laszlo and Federmeier, 2009); emojis (Weissman et al., 2024); pictures (Coco et al., 2020); and more. As such, it is considered by most as a domain-general, multi-modal index of the ease of semantic access, such that reduced N400s reflect facilitation in long-term memory access (Federmeier, 2022). Critically, N400 amplitude inversely relates to cloze probability, regardless of contextual constraint.^5^ ERP work as early as the 1980s has shown readers are sensitive to semantic context at the N400 (Kutas and Hillyard, 1980, 1984; Kutas, 1993; Kutas et al., 2006).

N400 effects have been argued as compatible with PC models, such that amplitude directly reflects lexicosemantic prediction error (Nour Eddine et al., 2022, 2024).^6^ Facilitated processing for predictable words translates to how easily the computational model “settles on a particular target representation” in the N400 time-window (Nour Eddine et al., 2022, p. 171). Specifically, expected inputs should decrease error unit activity, which theoretically represent smaller post-synaptic potentials (and thus attenuated N400 amplitudes). In general, PC captures N400 effects well since expectancy reduces N400 amplitude. Nour Eddine et al. (2024) successfully simulated a wide variety of N400 effects including isolated word recognition, non-word stimuli and sentence-level context effects. Of relevance, they simulated graded context effects for both orthographic and semantic manipulations. Importantly however, correlations between model simulations of cognitive tasks and brain activity do not imply that they are doing the same thing (Tuckute et al., 2024).

##### 3.1.1.2 Related information activated by strong contexts facilitates processing of anomalous words

A defining piece of evidence in favor of PC models is simulated semantic pre-activation in long-term memory reflected in the N400. A now well-known example of N400 category context effects, Federmeier and Kutas (1999) presented sentence pairs with expected category exemplars, within-category violations, and between-category violations (e.g., *Justin put a second house on Park Place. He and his sister often spent hours playing* monopoly/chess/baseball; target words underlined). The N400 effect to within-category violations (*chess*) was smaller than the N400 effect to between-category violations (*baseball*), despite these words being equally implausible. These kinds of “related anomaly” effects are highly reliable and are found across representational levels, including orthographic, phonological, semantic, and event-knowledge pre-activation (e.g., Kutas and Hillyard, 1984; Metusalem et al., 2012; Thornhill and Van Petten, 2012; Wlotko and Federmeier, 2015; Van Petten et al., 1999; Silcox and Payne, 2025).

These studies highlight the role of long-term memory in prediction, such that related information gets activated by strong contexts. While one could conceptualize this co-activation as an implicit feature of top-down expectations, it does not initially seem tenable that there would be *smaller* prediction errors generated (assuming that's what the N400 reflects) for *mispredicted* bottom-up input, simply because it overlaps conceptually (or structurally) with expectations. In other words, it seems counterintuitive to assume that orthographic, phonological, semantic, or event-related activation for an unexpected word would *not* show a robust prediction error response. However, Nour Eddine et al. (2024) were the first to successfully simulate these effects of anticipatory semantic overlap, and they argue this is exactly what is happening. They suggest there is *facilitated* lexical activation, such that both lexical and semantic prediction errors are suppressed from the corresponding top-down reconstructions. That is, N400 attenuation for within-category violations requires semantic overlap between predictions and sensory input, and lexical overlap, what they call “top-down lexico-semantic facilitation” (Nour Eddine et al., 2024, p. 16).

N400 interaction effects, such as whether orthographic neighborhood interacts with predictability, remain open to reconciliation within the PC framework. For instance, Nour Eddine et al. (2024) implemented PC model of the N400 simulated an interaction between orthographic neighborhood (ON) and contextual predictability (smaller ON effect as context increased). Few studies have directly tested this hypothesis. One line of work from Payne et al. (2015) and Payne and Federmeier (2018) has indirectly examined such interactions through the classic *word position* effect (Van Petten and Kutas, 1991; Dambacher et al., 2006), whereby N400 amplitudes to open-class words show a monotonic decrease in amplitude with linear word order within a sentence. Critically, this effect is only found for congruent meaningful sentences and is absent for random word strings or so-called “syntactic prose” sentences (coherent, grammatically ordered sequences of words that lack a coherent message-level semantic representation). Critically, while word position suppresses frequency effects on the N400 (consistent with predictive coding), these studies fail to find an interaction with orthographic neighborhood. These findings are also consistent with (Laszlo and Federmeier (2009)), who argued that neighborhood effects reflect the semantic system's inherent structural organization, such that visual word recognition is shaped by the similarity of orthographic inputs. Because of this structural constraint, such effects should remain even when strongly constraining contexts afford top-down predictions.

Nour Eddine et al. (2024) argued that the lack of interaction in these studies was likely due to the Stimulus Onset Asynchrony (SOA) in their studies of 500 ms [which is a faster SOA than where form-related prediction effects are observed in Ito et al. (2016a)], along with the presence of items that disincentivize prediction (random word strings and syntactic prose sentences). Moreover, another possibility is that the word position effect is too indirect a proxy of predictability. Payne and Federmeier (2019) addressed each of these concerns by adopting a participant-paced presentation rate, using only plausible sentences, and directly testing an interaction between orthographic neighborhood and predictability (via cloze probability). Here, they replicate the null predictability×ONinteraction (despite finding such an interaction with word frequency), conceptually replicating Payne et al. (2015). Nevertheless, this study also relied on a correlational design (i.e., exploiting single-trial variation in orthographic neighborhood of target words). An experiment orthogonally manipulating ON and predictability would be a valuable future contribution to the literature. There were also several arguably key effects that Nour Eddine et al. (2024) did not simulate, such as temporary semantic illusions (role reversal anomalies). These anomalies do not reliably elicit N400 amplitude modulation (Kuperberg et al., 2003; Kuperberg, 2007; Kim and Osterhout, 2005; Kolk et al., 2003), but likely do violate predictions. There have been mixed interpretations for whether these are reconcilable with PC frameworks (Bornkessel-Schlesewsky and Schlesewsky, 2019; c.f., Kuperberg, 2016) and remains an open question both empirically and for simulation work.

##### 3.1.1.3 Left anterior anticipatory negativity is strongest in highly constraining contexts

Electrophysiological research on neural activity *preceding* target words suggests that slow negative potentials may be implicated in predictive processing (for a review see León-Cabrera et al., 2024). For example, Li et al. (2017, in Chinese) showed at the pre-target interval (a few words before the sentence-final word), a left anterior sustained negativity was elicited strongest by high constraint contexts (lasting ~1,000 ms). While expectation suppression is a clear hypothesized effect for cases of *presented* expected input, (to our knowledge) there are no clear hypothesized effects for *not yet presented* expected input (c.f., Kok and de Lange, 2015). That is, most PC accounts are not unified on what is going on prior to stimulus onset. Identifying a clear hypothesis will serve as a critical link to foundational psycholinguistic theorizing on what should count as *genuine* evidence for prediction. For instance, some theoretical frameworks (e.g., post-lexical integration) place the criteria for prediction, or *anticipatory processing* based on contextual information, as needing to occur strictly *before* critical condition information (e.g., target word), and that this be a generative (active) process and not be observed only under unusual experimental conditions (e.g., very slow presentation rates; Pickering and Gambi, 2018; Ferreira and Chantavarin, 2018; DeLong et al., 2014b). Existing literature in vision on pre-stimulus sensory preparation and attention may be relevant here for anticipatory negativities (Kok et al., 2017; Barne et al., 2022), a discussion we return to in Section 3.3.3. For instance, in a perceptual decision task, pre-stimulus ongoing activity was hypothesized to reflect precise prediction errors (Hesselmann et al., 2010). Another possibility is that this sustained activity reflects top-down activity that gets elicited by predictions, a point we will return to in Hypothesis 4 when discussing the frontal negativity to expected words (e.g., Lai et al., 2024).

##### 3.1.1.4 Effects of prediction preceding predictable targets

Several RSVP studies demonstrate article-elicited graded N400 effects across multiple languages (also called the “pre-nominal prediction effect”; DeLong et al., 2005; Wicha et al., 2004; Van Berkum et al., 2005; Martin et al., 2013). The most famous example in English is the study by DeLong et al. (2005) using stimuli consisting of sentence contexts (e.g., *The day was breezy so the boy went outside to fly*…) with relatively expected (*a kite*) and unexpected (*an airplane*) article/noun pairs. They showed that N400 amplitude decreased (was less negative) as cloze probability increased, at both the article and the noun (see also a re-analysis of these data in Urbach et al., 2020). However, some studies have questioned the replicability of these findings in English (e.g., Ito et al., 2016b; Nieuwland et al., 2018, but see Yan et al., 2017).^7^ For example, Ito et al. (2016b) found no evidence for differential expectancy-based N400 effects at the article. However, they included implausible/ungrammatical filler sentences, which may have rendered readers unable to use the article as a reliable cue, a discussion that continues in Section 3.4.3 (see also Rabovsky, 2020 for related simulation evidence of this interpretation). Similarly, Nieuwland et al. (2018) failed to replicate DeLong et al. (2005) in a large multi-lab replication-attempt study. However, there were important methodological and analytical differences with their replication attempt, which may limit the reliability of their findings (Yan et al., 2017; Urbach et al., 2020; Nicenboim et al., 2020).

These article effects have been successfully simulated by Rabovsky (2020) in a change-in-state framework, such that congruent articles induce a smaller change in the hidden state of the network. This framework has conceptual overlap with PC and has explicitly been interpreted as a smaller prediction error (Rabovsky et al., 2018; see Nour Eddine et al., 2022 for a discussion). Other recent work using representational similarity analyses shows increased similarity between preceding and expected words in constraining contexts (Hubbard and Federmeier, 2021). These prediction effects preceding expected targets have been taken as strong evidence for anticipatory processing in reading (since they appear before bottom-up input) and are compatible with a PC account of the N400 (e.g., N400 reductions at the preceding article reflect reduced lexicosemantic prediction error). However, given the clearly small effect size and challenges with replicating this effect, more empirical evidence on the eliciting conditions of phonological form-based predictions is needed.

##### 3.1.1.5 Contextually graded N400 effects at parafoveal viewing

Similar to effects elicited at pre-target words in RSVP, evidence shows contextually graded N400 effects at pre-target words in both flanker RSVP paradigms (where the expected word appears parafoveally) and time-locked to the pre-target word in natural reading (for a recent review, see Schotter and Payne, 2026). Specifically, high-constraint (HC) contexts with expected target words facilitate (reduce) parafoveal N400 amplitude compared to low-constraint (LC) contexts (Stites et al., 2017; Barber et al., 2011). Furthermore, HC expected targets elicit smaller parafoveal N400s compared to HC unexpected targets^8^ (Li et al., 2023; Payne and Federmeier, 2017; Payne et al., 2019), an effect that has been recently replicated and extended to naturalistic sentence reading via co-registration of EEG and eye-tracking (Li et al., 2024; Copeland and Payne, 2025; Antúnez et al., 2022). Just like article-elicited effects, parafoveal N400 effects are consistent with PC if you take N400 amplitude to reflect prediction error, since amplitude scales with expectation. These findings are particularly relevant to natural reading, suggesting that the processes reflected in prediction error minimization may occur in advance of foveation.

#### 3.1.2 Repetition suppression

Repetition suppression is a robust phenomenon whereby neural activity is reduced for repeated stimuli (Auksztulewicz and Friston, 2016).

##### 3.1.2.1 Expectancy and (pseudo-)repetition

In single-word processing, the N400 consistently elicits repetition suppression, such that N400 amplitude is reduced when stimuli are repeated (Kutas and Federmeier, 2011). This work on repetition priming has been extended to evaluating whether there are downstream consequences to *mispredicting* in constraining contexts. Lai et al. (2021) observed graded N400 effects for sentence-final *unexpected* targets in strongly and weakly constraining sentences (at initial presentation). This critical target word was repeated after three filler sentences, and there were no repetition effect differences for prediction violations compared to equally unexpected targets in less constraining contexts (HC unexpected vs. LC unexpected).

In contrast, *expected* targets in HC contexts show evidence for *reduced* N400 attenuation at repetitions compared to unexpected targets (i.e., repetition effect was smaller for predictable words; Rommers and Federmeier, 2018a). Furthermore, when the critical “repetition” was an *expected* (but not actually previously presented) word for HC unexpected target initial sentences, (Rommers and Federmeier 2018b) observed this same N400 repetition suppression (pseudo-repetition effects; although these are not quite as strong as real repetition effects). In summary, expected words elicit *reduced* N400 repetition effects compared to unexpected targets, and also elicit so-called “pseudo-repetition” effects (i.e., targets show reduced amplitudes at first viewing if they were previously predicted but never shown; Lai et al., 2021; Rommers and Federmeier, 2018b).

Some hypothesize that in a PC framework, (real) repetition × predictability effects should be *larger* for more expected words, such that we should see expectation suppression for repeated stimuli (Song et al., 2024; Feuerriegel et al., 2021). In this interpretation, smaller repetition effects for expected words appears *inconsistent* with this hypothesis. However, one possible explanation is that sensory processing may be downregulated when uncertainty is low, creating weaker memory representations for predictable stimuli (Rommers and Federmeier, 2018a). Considering this interpretation under PC, in expected cases, prediction error was initially low, meaning the inference process was likely able to converge more quickly (compared to cases where prediction error was higher with unexpected targets), in which case this effect could be compatible with PC.

Most interpretations link these suppression effects to adaptation or learning mechanisms, which are computational-level goals of predictive coding. Two other computational models with prediction error frameworks (not predictive coding) have simulated these expectancy-based repetition effects (Fitz and Chang, 2019 and Rabovsky et al., 2018; for a detailed discussion of the differences between these models, see Nour Eddine et al., 2022, Figure 2). For instance, in the Error Propagation Model (Fitz and Chang, 2019), larger predictions errors increase weight updates (learning), such that unpredictable words elicit larger prediction errors at initial presentation, which create stronger predictions at repetition (resulting in larger repetition suppression for unexpected words). An important caveat of these error-based learning models is that they do not actually infer semantic information, but rather merely predict lexical items (Nour Eddine et al., 2022). Nour Eddine et al. (2024) were not able to simulate this interaction between predictability and repetition (although they do simulate other repetition and priming effects), which they argue is because their implementation did not include a learning mechanism that updates connection weights (but see recent models that have incorporated learning, e.g., Ohams et al., 2026).

Pseudo-repetition effects are less clear in how they fit within a PC framework. It could be that since the expected word was included in initial top-down predictions, it is still active in the state units and therefore attenuated (Lai et al., 2021). However, given that prediction error would be higher at initial presentation of unexpected words (and the top-down representation would presumably be weighted lower), this interpretation does not seem tenable within a PC framework. Another possibility is that they represent in-context learning, such that precision weighting is noisier for unpredicted words, producing a larger change for unexpected words compared to expected. However, this still does not fully account for why real repetitions produce larger effects than pseudo-repetitions.

Given that linguistic cases of repetition suppression can be considered to reflect a feature of memory, perhaps through learning, future work will need to explain how prediction error minimization interacts with memory representations beyond initial prior expectations drawn from long-term memory. This is a broader issue in computational modeling (i.e., not unique to predictive coding frameworks; Millidge et al., 2021; Futrell et al., 2020) based upon work in the neurobiology of memory on cortico-hippocampal interactions (e.g., Butola et al., 2025). Recent work combining expectation-based (but not PC) and memory-based models of sentence processing (Futrell et al., 2020) employing the principle of information locality may prove fruitful toward predictive coding architecture. For example, some emerging work in PC has started to make these links to memory via local prior predictions (Fountas et al., 2022). It therefore remains an open question whether PC architecture can simulate these expectation and longer-term repetition and pseudo-repetition effects.

##### 3.1.2.2 Parafoveal N400 is reduced upon foveation

Parafoveal N400 context effects are attenuated at target fixation (i.e., reduced target N400 amplitude for valid parafoveal preview; Stites et al., 2017; Payne et al., 2019; Milligan et al., 2023; Copeland and Payne, 2025). These could be considered a kind of repetition suppression effect, such that a target fixation is a repetition of what was previewed in parafoveal vision (Grainger et al., 2016; Mirault et al., 2020; Payne et al., 2019). These effects appear consistent with PC, given that the reader has presumably already accessed lexicosemantic information when they make a subsequent target fixation (and at least partially minimized prediction error by this point), their neural activity is reduced because there's no new unpredicted information to accommodate. Furthermore, although replicated many times, this foveal reduction of N400 amplitude after a parafoveal N400, is not always observed (e.g., Barber et al., 2011). Whether such foveal attenuation occurs may be related to the consistency of the preview as a reliable cue (e.g., use of pseudowords as preview). This has implications for PC architecture, such that this kind of repetition suppression may be dependent on the stimuli used, and perhaps even the broader linguistic environment, a point we elaborate in Section 3.4.3.

#### 3.1.3 Summary

Hypothesis 1 focuses on prediction error, such that error-signaling neural responses to sensory stimuli should scale inversely with expectation. N400 context effects, including anticipatory semantic overlap, parafoveal activation and foveal attenuation, are all largely consistent with PC models. Form-based activation at articles, the relationship between predictability and memory with predicted word repetition and pseudo-repetition effects, and the null interaction between orthographic neighborhood and predictability remain open questions for PC architecture, largely due to limited and/or conflicting empirical evidence. Furthermore, it is unclear whether or how reported anticipatory negativities in constraining contexts fit within PC.

### 3.2 Hypothesis 2 (Prediction): Top-Down signals represent sensory prediction

In PC models, top-down predictions from the generative model are transmitted down the cortical hierarchy, which are refined at each level (via state updating). In other words, top-down signals represent prediction itself, as contrasted with prediction *error*, as discussed in Hypothesis 1. Below, we report on three lines of research relevant to this hypothesis.

#### 3.2.1 Behavioral indices of predictability benefits

It is challenging to link behavioral responses to underlying neural mechanisms or specific stages of processing (i.e., sensory-specific effects). However, emerging work on PC in language processing has attempted to account for such effects. For example, Nour Eddine et al. (2024) suggest that “the time it takes for a model to settle on an input's correct lexical representation determines behavioral response times” (Nour Eddine et al., 2024, p. 17). To be consistent with their framework, behavioral responses should not reflect prediction error, but instead reflect internal state representations. This interpretation is similar to evidence accumulation models, such as the drift diffusion model, where response times reflect (in part) a product of the accumulation of evidence toward a decision threshold (Ratcliff et al., 2016), which, in language, may reflect a lexical threshold for word identification (Schotter, 2018; Schotter and Payne, 2026).

Robust evidence of rapid behavioral predictability effects has been observed in eye-tracking (ET) studies of reading. Early work demonstrated a few central, highly replicated effects of predictability. These include: (1) predictable words are skipped more often than unpredictable words, (2) when predictable words are fixated, fixation durations are shorter, and (3) predictable words provide greater parafoveal preview benefits (e.g., Ehrlich and Rayner, 1981; Inhoff, 1984; Balota et al., 1985; Drieghe et al., 2005; Rayner and Well, 1996). In general, behavioral facilitation appears consistent with PC, given that expectation does facilitate fixation durations. The idea of a lexical threshold driving eye movements is largely consistent with existing literature (Schotter, 2018; Schotter and Payne, 2026). Moreover, computational models of eye-movement control during reading such as E-Z Reader (Reichle, 2011) suggest that predictability effects influence the earliest stages of lexical processing. A pertinent finding is that predictability benefits both first-pass *and* second-pass eye movement measures (e.g., Luke and Christianson, 2016; Frisson et al., 2017, although second-pass effects are less reliable). If only a single lexical threshold drives fixation decisions, it is unclear why there would be downstream or “spillover” effects of predictability. However, since PC models implicitly account for multiple scales, it is possible that first-pass effects reflect lexical state activation, while second-pass effects relate to post-lexical (e.g., semantic or conceptual) state activation (consistent with interpretations such as Wong et al., 2025). Furthermore, predictive coding is compatible with a fully cascaded/interactive architecture (meaning it is not dependent on a threshold), and as such is not constrained to a staged access view of lexicosemantic processing. Though compatible, how such architecture maps onto known neurophysiological constraints, as well as what the implementation looks like, remains an open question (Millidge et al., 2021). As mentioned in Section 1.1.1, whether predictability effects are indeed dissociable along stage lines remains an ongoing question in psycholinguistics.

#### 3.2.2 Lack of prediction costs on reading behavior?

Behaviorally, there is a limited role of contextual constraint when processing unexpected input, even in highly constraining contexts. For instance, Luke and Christianson (2016) using corpus texts showed that lexical competitors (i.e., there is a more expected/higher cloze word), were facilitative rather than inhibitory. They also observed predictability effects for both first-pass and later measures, a nice demonstration in weakly constraining contexts of previously mentioned “later” effects. However, casting doubt on the ubiquity of null findings for behavioral prediction costs, Wong et al. (2024) showed prediction costs in the eye-movement record when anomalies were not present and two self-paced reading studies have shown evidence of prediction costs in a subset of the slowest trials (Payne and Federmeier, 2017; Jongman et al., 2023).

Continuing with the idea that behavioral response times reflect state unit activation, we conceptualize null prediction violation costs as state *stability*. In PC models, state units at any level reflect the previous iteration's top-down reconstruction and the incoming prediction error. It's possible that plausible prediction violations do not elicit a large enough immediate state change (either from top-down or bottom-up error) to impact lexical threshold accumulation, and therefore do not affect fixation durations (which we know are primarily driven by prelexical and lexical information). This may be consistent with other interpretations, such that the lack of prediction costs reflects a feature of probabilistic inference (Staub, 2025). However, empirical work suggesting a role for linguistic environment complicates this explanation (e.g., Wong et al., 2024), as well as work examining trial-level differences (e.g., Payne and Federmeier, 2017; Jongman et al., 2023).

#### 3.2.3 Relationship with word form

Nour Eddine et al. (2024) hypothesize that orthographic (without conceptual) overlap with a preactivated lexical representation (e.g., *step-stop*) should generate decoupled behavioral and electrophysiological responses: attenuated (less negative) N400 effects but longer response times. Behaviorally, orthographically related words provide smaller predictability benefits than expected previews, but invalid previews eliminate such predictability effects (Balota et al., 1985; Staub and Goddard, 2019). Electrophysiological evidence remains mixed on the precise relationship between word form and meaning activation, but most evidence suggests that they are similarly activated in the N400 (e.g., DeLong et al., 2019; Laszlo and Federmeier, 2009).

Milligan and Schotter (2025) co-registered eye-movements and EEG using a boundary change design with parafoveal preview words that were either expected (i.e., valid, identical preview) or anomalous orthographic neighbors of the most expected word (i.e., ON preview). The hypothesized relationship from PC models accurately predicts some of their observations—there was no difference between expected previews and ON previews on parafoveal N400 amplitude, but target word single fixation durations were longer for ON compared to expected previews. This pattern held only in cases where the reader fixated the target word (see also Nestor et al., 2025; Milligan and Schotter, 2024). In cases where the reader *skipped* the target word (i.e., only received parafoveal preview of ON), the parafoveal N400 was larger for ON, inconsistent with PC model predictions since the hypothesized effect was facilitation for ON. Therefore, for these target-skipped cases, PC models are not capturing the full range of effect dynamics due to a behavior-contingency (i.e., different pattern based on target skip decision). Additionally, these target-skipped trials are most akin to non-boundary change natural reading cases (where the preview does not change), which should be the intended relationship to model. However, it is difficult to parse the influence of predictability and plausibility here, since ON are contextually anomalous.^9^

#### 3.2.4 Omission responses

An omission response is neural activity produced in response to an expected stimulus that was never presented (for a review in auditory cortex, see Heilbron and Chait, 2018; see also Yaron et al., 2026). Omission responses should reflect top-down predictions, since there is no bottom-up input to elicit prediction errors (SanMiguel et al., 2013). No electrophysiology work with text-based stimuli (to our knowledge) has explicitly examined omission responses (for coverage of the omission paradigm, see Braga and Schönwiesner, 2022). The closest paradigm is Schotter et al. (2023) orthogonal masking study using flanker-ERP, where the double mask condition (parafoveal and foveal mask) could be considered an “omission.” They showed that the N400 effect was eliminated in this case, potentially inconsistent with the hypothesized effect in PC.

We therefore discuss relevant behavioral evidence. Following Nour Eddine et al.'s (2022) interpretation that behavioral evidence represents *state unit* activation, then omission responses should be translatable to behavioral evidence, such that state activation would exclusively reflect the influence of predictions (top-down bias), given the lack of bottom-up input with modulatory prediction error. There is some relevant eye-tracking reading work from Staub et al. (2019, 2024, 2025) on omission and repetition errors. However, for natural sentence reading there is no *true* omission of bottom-up input since the reader can always proceed forwards or backwards through the text.

Staub et al. (2024) presented readers with sentences containing function word repetitions or omissions. On average, readers missed both types of errors ~37% of the time, and this effect was slightly larger for more predictable function words. When readers *failed* to notice these errors, there was no disruption to eye-movements. However, when they *do* report an error, repetitions primarily influenced first-pass reading, while omissions primarily increased re-reading. They suggest top-down linguistic knowledge via perceptual inference explains this lapse in noticing errors (see also Huang and Staub, 2021). However, function words are processed differently than content words (e.g., readers notice noun repetitions ~90% of the time, Staub et al., 2019). With this design, PC model architecture predicts both an omission response and repetition suppression response. Omission responses here are not consistently observed—only in cases of conscious error detection —which appears *inconsistent* with PC models strictly, although it is difficult to say whether this design exactly mirrors the hypothesized effect since it is not a “true” omission (i.e., absence of all bottom-up input). In explicit noticing cases however, this pattern appears consistent with PC models, given that readers show longer go-past and total fixation times in the target region. Failure to notice repetitions with no behavioral disruption could be considered a case of *repetition suppression*. These effects may be modulated by precision-weighting, as there appears to be a reliability component to this effect: faster readers notice fewer errors (expect lower reliability; Staub et al., 2024). Specifically, the precision of the *bottom-up error* hypothetically is smaller in these faster reader cases. Alternatively, it could be the precision of the *top-down bias* that is larger, such that faster readers rely more on their higher-level representations than bottom-up input. Consistent with this idea, Payne and Federmeier (2019) showed that faster readers show *stronger* effects of predictability on the N400, which they interpreted as reflecting increased reliance on prior context. Additionally, future eye-tracking analyses on more naturalistic omission errors when reading assistive text captions of speech may be informative for this hypothesized response as such errors appear to strongly diminish the behavioral benefits of assistive text captioning (Crandell et al., 2022).

#### 3.2.5 Summary

Hypothesis 2 focuses on prediction, such that top-down signals represent sensory prediction. Assuming behavioral indices reflect state unit activation, multiple effects of predictability are consistent with this hypothesis. Neural responses that are contingent on a particular behavioral decision (i.e., that have a coupled relationship) appear partially consistent with PC (e.g., orthographic neighbor N400 interaction with target skip decision), since only one of the expected effects was explicitly hypothesized. It is unclear whether and how such real-time dynamics would be accounted for under this architecture. Similarly, omission responses to function words that are sensitive to attention modulation are not yet accounted for under PC architecture.

### 3.3 Hypothesis 3 (Biological Realism): At each level of the cortical hierarchy there are two functionally distinct neural subpopulations representing predictions and prediction errors

A good computational model should be able to satisfy biological or neural realism (Mazzoni et al., 1991). PC models predict there are separate sets of neurons across cortical layers for feedback predictions (state units) and feedforward prediction errors (error units), although there are diverging implementations in the “cortical hardware” (Huettig, 2025, p. 245). The most common proposal is that prediction errors are encoded by superficial pyramidal neurons (which are large contributors to EEG), while predictions are encoded by deep pyramidal cells, and these signals are carried by different frequency oscillations (e.g., Friston, 2005; Spratling, 2016, 2017).

#### 3.3.1 Localization of predictability

Nour Eddine et al. (2024) N400 PC model predicts that predictability effects on the N400 should localize to functionally distinct populations of error and state units. Prior empirical work suggests that lexico-semantic predictability effects are localized to left temporal regions, which support word processing (Wang et al., 2018b). Wang et al. (2023) showed in left ventral temporal regions, expected word processing was facilitated in left posterior occipitotemporal fusiform cortex and left mid-fusiform cortex, corresponding to orthographic and lexical facilitation, respectively. Processing was also facilitated in left lateral temporal regions, specifically left superior temporal cortex and left middle temporal cortex, corresponding to phonological and lexical facilitation (Wang et al., 2023). Facilitated processing in left medial temporal cortex and bilateral anterior ventral temporal poles may indicate the “reduced need to retrieve and ‘bind' distributed semantic features into distinct concepts.” (Wang et al., 2023, p. 4489). However, other evidence shows N400 effects generally recruit a widespread (but left-dominant) fronto-temporal network (e.g., Lau et al., 2008) and emerging work using TMS shows causal evidence for a selective role of left inferior frontal cortex in N400 predictability effects (e.g., Silcox et al., 2023). Taken together, predictable words facilitate activation in cortical areas relevant for inferring meaning from orthography, the central aim of PC (inferring meaning from sensory input). We turn now to evidence that these precise neuroanatomical sources are functionally dissociable as error and state units, consistent with PC.

Wang et al. (2025) present evidence for the biological plausibility of PC in language using multivariate analyses on MEG data. They tested two hypotheses from PC. First, for the N400, expected words will “reinstate pre-activated lexico-semantic predictions *within the same left ventromedial temporal region* that produces a larger evoked response to unexpected inputs” (Wang et al., 2025, p. 11, emphasis added). Specifically, they hypothesize expected words will produce consistent temporal patterns at both pre-target and target N400 time-windows *in the same region* as the increased N400 amplitude to unexpected words. They found support for prediction reinstatement, such that temporal patterns were more similar for expected than unexpected words. PC models predict that prediction signals (as reflected in state activity) should remain relatively stable, while error signals should decay rapidly (de Lange et al., 2018). Such similarity across pre and target windows for expected targets could reflect this kind of stability, but other work suggests that confirmed predictions lead to impoverished representations (e.g., Hubbard and Federmeier, 2021).

Wang et al. (2025) second hypothesis is that reinstatement of predictions should activate state units that are *distinct* from error units. This requires the assumption that voxel activation reflects a mixture of both state and error units, such that expected words should activate *state* units, and unexpected should activate *error* units. They found evidence that spatial patterns in the N400 time-window were more similar for matching condition pairs than expected-unexpected pairs (despite a reduced univariate response for expected words). Future work with improved spatial resolution will better elucidate whether such activity originates from the same or different sub-populations of neurons.

Taking a step back however, emerging work draws into question the prevalence of such neural units at all. For instance, Westerberg et al. (2025) applied neurophysiology and optogenetic methods to study visual cortex in mice and monkeys. They presented evidence from an oddball task that predictable oddballs were *robustly* signaled by feedforward spiking responses, while unpredictable oddballs were *weakly* signaled with limited feedback. They found no evidence for feedforward prediction errors in L2/L3 cortex. They interpret these findings as reflecting that PC is non-ubiquitous even within sensory cortex (see Gabhart et al., 2025 for an alternative model called predictive routing). Critically, if this kind of circuit interactions are not ubiquitous within sensory cortices (see also Westerberg and Roelfsema, 2026), they are especially unlikely to expand to association cortex, including language network(s) that are highly spatially distributed and cellularly/structurally heterogeneous.

#### 3.3.2 Cortical information flow across oscillatory frequencies

To support such functional dissociation of state and error units (potentially in the same neuroanatomical regions^10^), *neural oscillations* have been proposed as a mechanism by which information flows through the cortical hierarchy (i.e., capable of coordinating communication across spatial and temporal scales; Arnal and Giraud, 2012). Specifically, it has been proposed that top-down predictions are primarily carried by slow oscillations (alpha/beta), while bottom-up activity is carried by fast oscillations (gamma; e.g., Michalareas et al., 2016; Chao et al., 2018; for a brief overview see Giraud and Arnal, 2018). There are differing hypotheses about which specific oscillatory bands reflect top-down and bottom-up information flow, and whether and how these frequencies are coupled (e.g., Bastos et al., 2020). There is some relevant literature on oscillatory activity in language processing (for reviews see Lewis and Bastiaansen, 2015; Lewis et al., 2015), but studies explicitly designed to parse prediction from prediction errors are largely missing from the literature. For example, a recent PC model showed that recognition of syllables (parsed from continuous speech) was facilitated during cross-frequency theta-gamma coupling (Hovsepyan et al., 2020). Regev et al. (2024) showed three unique temporal profiles in the language network in the high-gamma range to different-sized linguistic units (words, short phrases, and sentences). They suggest these distinct profiles reflect temporal receptive windows, or the amount of preceding information that influences processing on the current word. These data support the existence of temporal hierarchical functional organization, such that linguistic information is carried in oscillatory frequencies hypothesized to be relevant for inferring meaning during sentence processing. Similarly, work using spoken narratives demonstrates unfolding predictions at multiple levels of representation across timescales, consistent with PC architecture (Caucheteux et al., 2023). However, work on cortical speech tracking suggests the signal may be dominated by transient evoked responses (Lalor and Nidiffer, 2025), as contrasted with the popular alternative of narrow-band oscillatory entrainment (for a relevant introduction to oscillation dynamics see Tavano et al., 2023). However, non-oscillatory activity (e.g., broadband change) is often unaccounted for in these studies, drawing into question the reliability of some conclusions (e.g., Donoghue et al., 2020; see Woods et al., 2024 for a concrete example). Taken together, emerging work suggests that the coordination of slow and fast oscillatory dynamics is critical for temporal binding during perceptual inference—a direct link toward a neural mechanism for optimizing communication timing for predictions. Oscillatory activity offers a unique advantage at tracking continuous processing to capture unfolding spatiotemporal dynamics, and indeed an emerging area for PC models aims to explicitly account for temporal predictions (Millidge et al., 2024; Ohams et al., 2026).

#### 3.3.3 Anticipatory alpha modulation

While alpha oscillations are thought to carry predictions (e.g., Mayer et al., 2015), the direct link between power modulations and PC mechanisms is less developed. In visual perception, pre-stimulus alpha power suppression (i.e., power decrease) has been reported when anticipating an upcoming visual stimulus (Bidet-Caulet et al., 2012; Jensen et al., 2012; Mathewson et al., 2014; Jensen, 2024). In language, a number of investigators have reported similar alpha suppression in strongly vs. weakly constraining contexts just prior to seeing an expected word (Rommers et al., 2017; Wang et al., 2018a; León-Cabrera et al., 2022). This effect has been interpreted to reflect anticipatory increases in cortical excitability, or preparation for sensory input (given the link between alpha and cortical inhibition; Jensen and Mazaheri, 2010). Just like in our discussion of anticipatory negativities in Section 3.1.1.3, there are not strong claims in PC about activation *prior* to stimulus onset. However, in highly constraining contexts that afford strong (i.e., lexical) predictions, the top-down predictions carry higher subjective certainty. Alpha power may therefore reflect the top-down precision weighting of sensory evidence (e.g., León-Cabrera et al., 2022; Hetenyi et al., 2025; Terporten et al., 2019; see Section 3.4 for a deeper discussion of evidence for precision weighting). In this interpretation, alpha suppression reflects an increase in local cortical excitability (i.e., reduced inhibition), acting as a kind of gain-control mechanism to limit the influence of prediction error (e.g., Kerlin et al., 2010). Given evidence linking slow-wave oscillations to top-down predictions described in the previous subsection, this kind of anticipatory suppression appears generally consistent with PC architecture. Specifically, such top-down oscillatory dynamics may be linked to faster state stabilization across hierarchical levels, such as signaling to upregulate predictions in constraining contexts. This mechanism serves to pre-activate the neural populations tuned to the predicted stimulus, effectively lowering the threshold for state stabilization across hierarchical levels. By pre-activating expected representations, the system can more rapidly resolve the mismatch between top-down expectations and bottom-up prediction error, minimizing the duration and magnitude of the prediction error signal.

#### 3.3.4 Summary

Hypothesis 3 states that PC models should be biologically plausible, such that at each level of the cortical hierarchy there are two functionally distinct neural subpopulations representing predictions and prediction errors. Predictability effects localized to left ventromedial temporal cortex are consistent with PC. One study showing such localization is spatially overlapping, yet functionally distinct for error units and state units is also consistent evidence. However, there remains much to be specified, such as how these signals are transmitted and whether higher-order laminar levels in left temporal cortex follow such specifications, especially given recent reports of absence of such effects even within sensory cortices (Westerberg et al., 2025; Westerberg and Roelfsema, 2026).

### 3.4 Hypothesis 4 (Hierarchical Inference): Prediction error minimization is achieved through reciprocal exchange of error and prediction signals across levels

Causal structure (environmental regularity) is encoded by our internal generative models and hierarchical levels. The hierarchical structure of PC (layers of prediction and error units) allows for interactive communication between higher and lower order levels. In other words, information flows in both directions, such that specific, local information informs inferences about global patterns, and vice versa. Therefore, an inherent feature of the hierarchical structure of PC models is context-dependence (e.g., expectations about the current situation). Furthermore, precision weighting of prediction errors allows the system to handle context-sensitive *uncertainty* (e.g., ambiguity, noise, or expectations about signal quality). To achieve prediction error minimization, there's a complex “interplay between top-down suppression of prediction error and bottom-up facilitation of prediction” such that “top-down activity elicits activity as it suppresses prediction error, and precision optimization works against the suppression and in turn elicits more top-down predictive activity” (Hohwy, 2013, p. 67). Put simply, reliability modulates the degree to which top-down vs. bottom-up signals are prioritized. For instance, when bottom-up precision is low, predictions are up-weighted and prediction errors are down-weighted (de Lange et al., 2018). We now turn to discussion of empirical evidence for hypothesized information exchange across hierarchical levels and precision-weighing in language processing.

#### 3.4.1 Context and task-dependence of the frontal positivity^11^

In contrast to robust effects of predictability on the N400 component, the eliciting conditions and functional interpretations of the frontal positivity are less consistent in the literature, and we therefore provide more literature synthesis here (Van Petten and Luka, 2012). The *frontal positivity* (also called anterior post-N400 positivity, late anterior positivity, anterior positivity, and late frontal positivity across the literature) is the most commonly elicited ERP component to *plausible* prediction violations (e.g., “She shut the front window and locked the back *room…”;* DeLong et al., 2014a), with typical spatiotemporal dimensions at anterior sites ~500–1,000 ms after stimulus onset (for a review see Van Petten and Luka, 2012). This frontal positivity is functionally dissociable from a late posterior positivity/LPC/P600 elicited by semantic anomalies (e.g., Brothers et al., 2020; Li et al., 2023; Kuperberg et al., 2020; Quante et al., 2018). In an early demonstration tying prediction to the frontal positivity, Federmeier et al. (2007) showed that plausible prediction violations (unexpected words in highly constraining contexts) uniquely elicited a frontal positivity. That this effect was present only for plausible prediction violations and not merely unexpected words (e.g., in low constraint sentences) provided compelling evidence that this effect was functionally related to revision-based processing of prediction violations (also called processing consequences or costs; Lai et al., 2024; Payne and Federmeier, 2017; Hubbard and Federmeier, 2021; Federmeier, 2022), which may entail suppressing a predicted but not observed lexical candidate (Ness and Meltzer-Asscher, 2018; Ryskin and Nieuwland, 2023). Other work has reported frontal effects that occur *invariant* of sentential constraint (i.e., unexpected words are more positive than expected words regardless of constraint), which has led to theories positing that the frontal positivity is driven by broader updating of a situation model, discussed below (Kuperberg et al., 2020; Brothers et al., 2020, 2023).

While some have found that global discourse context modulates the elicitation of a frontal positivity (Brothers et al., 2020; Kuperberg et al., 2020), others have found evidence for a frontal positivity to prediction violations even in minimal contexts (e.g., a category cue—*a type of tree;* Federmeier et al., 2010; Ryskin et al., 2020). Other work suggests the frontal positivity may be modulated by active task goals, such as being instructed to actively predict (Brothers et al., 2015). At the same time, others have failed to find effects of task or instruction effects (e.g., dual-task demands, top-down instructions to predict or read for plausibility) on the frontal positivity specifically to plausible prediction violations (Payne et al., 2019; Lai et al., 2024).

One framework for understanding late positivities suggests that they are indicative of change in the communicative environment (Kuperberg et al., 2020). The frontal positivity here reflects *successful* situation model updating (e.g., Kuperberg et al., 2020; Brothers et al., 2020, 2023). It is important to qualify the following discussion as *conceptual* application to predictive coding, as there is not yet (to our knowledge) an implemented predictive coding model characterizing late positivities from which to derive concrete predictions. In PC model terms, the frontal positivity could reflect new top-down bias (predictions) generated in situation model reconstructions. In other words, top-down bias would be propagated down from the higher-level situation model level to update semantic and lexical states (Wang et al., 2023). The difficulty in explaining the frontal positivity lies in specifying why this unit activity is unique to unexpected plausible words, and insensitive to implausible unexpected words. Wang et al. (2023) circumvent this issue by proposing that the LPC (elicited to anomalies) represents prediction error at a lower-level orthographic level (because top-down predictions did not suppress this low-level error), referred to as “destabilization of the lexico-semantic state” (Wang et al., 2023, p. 4493). This idea that the frontal positivity reflects top-down activity elicited to suppress lower-level prediction error is the only clear, tenable link to PC models. However, it remains unclear whether such activity necessarily needs to reference a change in a situation model.

To evaluate this proposal further, we discuss Lai et al. (2024) since their design crossed strongly and weakly constraining contexts with active (i.e., reading under explicit prediction goal; Brothers et al., 2015) vs. passive comprehension. They showed unique elicitation of the frontal positivity to highly constraining contexts with unexpected words (i.e., no difference between weakly constraining unexpected and weakly constraining expected, even when participants classified these as being prediction violations). The frontal positivity not reliably eliciting *graded* activity in response to unexpected input is the most difficult to reconcile with PC models (see also Federmeier et al., 2007; Wlotko et al., 2012; Payne and Federmeier, 2017). While Lai et al. (2024) findings are compatible with prior work showing the frontal positivity is only elicited by prediction violations, this is complicated by other studies that do show contextually graded activation, such as (Brothers et al. 2015, 2023). Moreover, Lai et al. (2024) showed when participants' predictions did not match the presented words, the frontal positivity was enhanced (unique to high constraint unexpected), which could be a kind of “graded” effect.

Lai et al. (2024) showed that active prediction did not strengthen the frontal positivity, although it did strengthen a frontal negativity to expected items (uniquely for exact matches). There is not consensus on whether the frontal positivity and frontal negativity are supported by the same underlying neurocognitive mechanisms. Existing evidence suggests that they may originate from different processes since they are elicited by different experimental conditions and show different sensitivities to individual differences and task effects (Lai et al., 2024; Federmeier, 2022). The frontal negativity has been linked to ambiguity-resolution processes and it is uniquely elicited by *expected* stimuli (while the frontal positivity is elicited by *unexpected* plausible stimuli, discussed at the start of Section 3.4.1). This frontal negativity, like the anticipatory negativity discussed in Hypothesis 1 could itself index top-down activity, such that strengthened predictions suppress prediction error, although with this interpretation it's unclear how both the frontal positivity *and* negativity could be represented by the same mechanisms. If we are to take these studies as evidence for the frontal positivity reflecting top-down error, then alternative explanations for state-like activation need to be causally attributed. Given that there is “no direct competition amongst activated lexical items” (i.e., “no lateral lexical-level inhibitory connections amongst state units”; Nour Eddine et al., 2024. p. 17, but see their footnote 10 on input suppression), we would expect that the frontal positivity should be similarly elicited in cases where there are more or fewer lexical competitors (note that this is distinct from earlier conceptions of the frontal negativity, which showed that its magnitude did scale with the number of high probability competitors/entropy; Wlotko et al., 2012). This hypothesis appears consistent with work by Brothers et al. (2023); however, other implementations allow for biased competition (e.g., Millidge et al., 2021). Additionally, as Millidge et al. (2021) broadly suggests for PC architecture, future work should consider whether there is an additional (or concurrent) attention-based precision-weighting mechanism, which may be implicated here for the frontal positivity. More empirical work is needed to specify the role of top-down goals in modulating linguistic processing (e.g., on sustained ERPs and its relationship to oculomotor decisions), as well as simulation work to test this alternative precision-weighting mechanism.

#### 3.4.2 Relationship with experience

Generative models are inherently formed by an individual's experiences. If the frontal positivity reflects top-down error, then this effect should be enhanced when bottom-up precision is low. It could be more precise in either of these ways: that bottom-up error is reduced or that top-down bias is larger. Work on individual differences suggests that this component follows more of a discrete pattern, which could be connected to better or worse precision. Older adults are not as sensitive to prediction violations in behavioral (eye movements: Andrews et al., 2023) or neural indices (delayed and reduced N400: Payne and Silcox, 2019; absent frontal positivity: Federmeier et al., 2010; Wlotko et al., 2012; DeLong et al., 2012). However, there appear to be interindividual differences in this, such as those with high verbal fluency (Federmeier et al., 2010; Dave et al., 2018; DeLong et al., 2012) showing frontal positivities to prediction violations. Further evidence shows that engagement in predictive processing may also vary *within an individual* from trial to trial (Jongman et al., 2023; Payne and Federmeier, 2017). Similar patterns have been observed in lower-literate adults (Ng et al., 2020), L2 language users (Ito et al., 2016b) and children (Zhao et al., 2019). This suggests there is some critical experience component to predictive processing. This remains an open question, and future work should evaluate whether text-based factors (e.g., stimulus quality as a proxy for precision) influence the presence and magnitude of the frontal positivity [as observed by Silcox et al. (2024) in auditory processing].

#### 3.4.3 Relationship with environment

One factor that may modulate predictability effects is the current global linguistic environment, or overall statistics of text materials, which appear to adaptively enhance or suppress propensity for prediction. Some differences in findings [e.g., article effects in DeLong et al. (2019) vs. Ito et al. (2016a) and DeLong et al. (2005) vs. Nieuwland et al. (2018)] may be attributable to differences in the broader materials of the experimental session. For instance, (Brothers et al. 2017, Exp 2) manipulated predictive validity with no semantic anomalies (proportion of high-cloze targets in HC fillers: all HC expected fillers, all HC unexpected fillers, or a mixture) and showed greater reading time facilitation for highly predictive contexts. Having a larger proportion of confirmed predictions led to enhanced predictability effects compared to when predictions were more often disconfirmed (see also Lau et al., 2013).

Readers may up-regulate predictions when they are consistently being confirmed, as Wong et al. (2024) suggest in their study showing that the presence of prediction violation costs depends on being in a plausible linguistic environment. They suggest lexical prediction is sensitive to overall environmental demands, such that readers may shift to a more top-down approach when only plausible sentences are presented. This is largely compatible with a precision-weighting mechanism that is sensitive to ongoing contextual regularities. While existing work comparing across studies provides a good starting point, future work should explicitly evaluate whether there are trial order effects across experimental sessions, such as whether predictions are enhanced in an incremental fashion when the stimuli continue being reliable (e.g., Delaney-Busch et al., 2019). This mechanism can also explain noisy visual signals, which can also be considered as unreliable cues. For instance, we would expect that the generative model decreases sensitivity to top-down signals, and thus reduces prediction and increases bottom-up signals. Some have attributed this sensitivity to linguistic environment to “rational adaptation” theory, such that readers dynamically adjust the *strength* of their predictions, rather than the presence (e.g., Kuperberg and Jaeger, 2016). It is “rational” in the sense that readers are predicting more when their predictions are confirmed, thereby adapting to the likelihood of success (Nieuwland M., 2021). However, there's mixed evidence for this hypothesis (e.g., van Wonderen and Nieuwland, 2023; Ness and Meltzer-Asscher, 2021; Nieuwland M., 2021; Nieuwland M. S., 2021; Delaney-Busch et al., 2019).

This so-called “predictive validity” may also be related to studies of parafoveal preview showing preview type differences. While valid previews provide parafoveal preview benefits, there also appear to be unique costs associated with invalid and visually degraded previews (e.g., Schroyens et al., 1999; Vasilev et al., 2018; Andrews and Veldre, 2019). For example, Hutzler et al. (2013) demonstrated X-masks interfere with foveal word recognition, a finding that can be extended to other kinds of non-valid previews. It is possible that real-time, dynamic precision weighting is down-regulating predictions and up-regulating the bottom-up input in these cases, which could explain why readers are so sensitive to upcoming illicit information. The mere presence of mismatched (invalid) preview information likely makes it more difficult to converge on an appropriate inference. Although some work suggests no early relationship between stimulus quality and predictability (e.g., Staub, 2020), future work should evaluate whether readers are *more* sensitive to perceptual noise/boundary changes in cases of poor overall linguistic reliability vs. high reliability (e.g., cases where all sentences are high-constraint expected). Precision can also potentially be evaluated by directly comparing mispredicted (high precision) and unpredicted (low precision) conditions (e.g., Hsu et al., 2018). For instance, N400 amplitudes to anomalous words during false hearing (i.e., high confidence auditory misperception of an anomalous phonological neighbor as an expected word) in noisy speech perception is similarly reduced as when actually perceiving an expected word (Silcox and Payne, 2025).

#### 3.4.4 Summary

Hypothesis 4 focuses on hierarchical inference, such that prediction error minimization is achieved through reciprocal exchange of error and prediction signals across levels. These signals are modulated by precision-weighting. Evidence showing null prediction violation costs in eye-tracking (in some cases), sensitivity to linguistic environment plausibility, and preview benefits and costs can all seemingly be accounted for under a PC framework. While the frontal positivity can be argued to reflect top-down bias, that it is not reliably elicited, nor always in a graded fashion, nor differentiated in this model from the frontal negativity are difficult to reconcile with PC. Future work will be able to directly test some of these open hypotheses, such as whether the frontal positivity is routinely generated or specific to prediction violations, or whether there is a relationship with stimulus quality.

## 4 Challenges and future directions

There exists a rich historical tradition in cognitive science of connectionist frameworks/neural network models being developed to explain the dynamics of cognitive processing (Kriegeskorte and Douglas, 2018). Semantic processing has had a privileged place within this general endeavor (e.g., Parallel Distributed Processing, Rogers and McClelland, 2004), and it seems only fitting that predictive coding architecture applications to language would evolve from this tradition. Computational modeling, therefore, is a highly valuable endeavor that should continue to exist in tandem with hypothesis-motivated empirical research (Guest and Martin, 2021). Such a multifaceted approach will bring us closer toward refining our theories of linguistic processing, therefore making it possible to adjudicate between otherwise difficult to interpret data or intuitive explanations.

A critical challenge with PC is that it is inherently difficult to falsify. This is due to several reasons, including (but not limited to) underspecification of neurocognitive mechanisms, difficulty evaluating compatibility of empirical evidence for predictive coding, lack of critical distinguishing empirical evidence for biological plausibility, generalizability to all sensory-cognitive processing, and representation and causality in neural coding. As an example, the precision-weighting mechanism can simultaneously elicit or suppress neural activity based on the context (Huettig, 2025). This makes it difficult to infer whether any individual experimental effect supports top-down or bottom-up activity, and one could argue for whichever side best fits an existing perspective (Huettig, 2025). A related issue is the assumption that any evidence for *prediction* supports *PC*. This is a categorical mistake, since PC is a model of neural efficiency, not some broad framework (like the free energy principle; Hohwy, 2013). Similarly, (Sprevak 2024) suggests that accepting any (or all) concepts related to PC “does not constitute an endorsement of PC,” nor does evidence for these ideas necessarily reflect unequivocal evidence for such neural architecture (p. 8).

If one is unwilling to accept prediction error minimization as the unified and complete explanation for what the brain does, or even the one cognitive feat of language, one can imagine a more accommodative or flexible view of predictive coding architecture. For instance, it is entirely possible that predictive coding is just one mechanism supporting language processing, in specific functions (e.g., lexico-semantic error representing N400 amplitude) or neural circuits, but it need not be taken as the central architecture of all language processing (or cognition more generally). For example, the Glenmore model of eye movement control (Reilly and Radach, 2003) implements the Interactive-Activation (IA) model of word identification (a connectionist framework), despite IA being limited in scope independently [as discussed in Reichle (2021)]. In this same way, PC architecture may be able to “slot” into existing computational models of linguistic processing to expand explanatory power, particularly for models which otherwise have opaque neurobiological links. This point has been made elsewhere about PC models, such as Millidge et al.'s (2021) speculation that the field will likely move toward heterarchical (i.e., non-hierarchical) and specialized architectures, and (to a degree) by (Federmeier 2022) in the discussion that the N400 does not directly reflect prediction error *per se*, since prediction is just one of the factors influencing semantic access, and thus N400 amplitude.

Given the relative infancy of PC applications to language, it is unsurprising (minimized prediction error?) that there are underspecified or incomplete aspects. Critically, PC must be mechanistically specified at the neuronal level (e.g., which neurons in left ventromedial temporal cortex represent error units?). There are also underspecified connections to other cognitive functions (e.g., how does perceptual processing of text relate to long-term memory and downstream consequences of prediction error?). Additionally, many discussed predictability effects can be explained by other models (e.g., Nour Eddine et al., 2022). For instance, effects of cloze probability on the N400 have been simulated by other computational models without prediction error mechanisms (e.g., Retrieval-Integration, Brouwer et al., 2017).

Throughout this discussion, future directions were offered where appropriate (e.g., sub-hypotheses for the precision-weighting mechanism). A key area of empirical study not covered in this analysis concerns how implausibility or semantic anomalies fit within a PC framework. While we covered some work on *related* anomalies (see Section 3.1.1.2 and 3.2.3), a critical application space will be work on the downstream consequences of implausible words, such as re-reading and the late positive component (LPC). For instance, *plausibility preview benefits* (i.e., facilitation for plausible vs. implausible previews) are elicited rapidly during initial (first-pass) stages of processing (e.g., parafoveal N400, gaze duration) and may be transient (e.g., Schotter and Jia, 2016; Veldre and Andrews, 2016, 2017, 2018; Li et al., 2024). However, plausibility of the foveal (target) word can independently modulate the foveal N400, and implausible targets elicit an LPC, an effect not observed parafoveally (e.g., Li et al., 2022, 2024; Schotter et al., 2023). Furthermore, some work has linked the LPC in natural reading to regression decisions (Metzner et al., 2017). While we discussed briefly parafoveal processing (see Section 3.1.2.2 and 3.4.3), the ongoing, temporally distributed dynamics between parafoveal and foveal representations across saccade behaviors remain yet to be accounted for under a PC framework (see Schotter and Payne, 2026 for a discussion of the importance of brain-behavior coupling approaches).

A broader future direction will be to explore whether other cognitive domains (e.g., event cognition, music perception, speech perception) are explainable by PC, consistent with PC as a multimodal, domain-general explanation for neurocognitive processing. This is consistent with increasingly prominent trends toward multi-method approaches to bridge often disparate theoretical domains and traditions, and studies examining domain-generality of phenomena (e.g., Võ and Wolfe, 2013; Fennell et al., 2021). For example, Schotter et al. (2025) argue utilizing co-registration of eye-tracking and EEG/MEG can bridge diverse visual attention literatures across multiple domains, including scene perception, face perception, reading, and visual search.

## 5 Conclusions

Following Walsh et al. (2020) review of PC applications in visual cognition, we explored four PC hypotheses in their application to language processing: bottom-up prediction error, top-down predictions, biological plausibility, and hierarchical inference. Across hypotheses, we argued that the following effects are compatible with PC models (see also Table 1): (H1) N400 context effects, anticipatory semantic overlap, parafoveal-to-foveal attenuation, (H2) behavioral indices of facilitated recognition, first-pass and second-pass effects of predictability, (H3) localization to left ventromedial temporal cortex, spatially overlapping, yet functionally distinct localization for error units and state units, and alpha suppression, (H4) null prediction violation costs in eye-tracking, sensitivity to linguistic environment plausibility, and preview benefits and costs.

Some predictability effects showed consistency with PC hypotheses for a particular behavior, but were violated if that behavior changed, and were therefore categorized as partially consistent: (H2) behavioral coupling with neural responses (orthographic neighbor N400 interaction with target skip decision) and variably evoked omission response to function words. Other effects were more difficult to reconcile with a PC framework currently and/or require more empirical evidence: (H1) activation at articles, predicted word repetition and pseudo-repetition effects, null interactions between orthographic neighborhood and predictability, left anticipatory negativities, and (H4) the frontal positivity, which could reflect top-down bias, but it is not reliably elicited in a graded fashion, nor differentiated in this model from the frontal negativity to expected words.

PC models have strong appeal because they offer a parsimonious explanation of neurocognitive processing and offer inroads to connect language processing with a broader literature in cognitive and sensory neuroscience. While PC does provide compelling explanations for several emerging linguistic phenomena, it may not capture the dynamics of *all* language processing. An interesting trend is emerging in visual neuroscience suggesting limitations to the ubiquitous application of PC theory (Westerberg et al., 2025; for reviews see Gabhart et al., 2025 and Westerberg and Roelfsema, 2026). In other words, PC could plausibly be one of the many mechanisms that support language comprehension, but it does not necessarily need to reflect the core architecture of the language system.

## Footnotes

1 The same general patterns are observed regardless of how predictability is operationalized. Other common methods include estimates derived from corpus data, statistical and large language models (e.g., transitional probability, surprisal and entropy).

2 As an aside, some have (controversially) argued that generative models have fundamental conceptual issues with representation and causality in neural coding, a general term describing the (Shannon) communication metaphor in neuroscience (Brette, 2019; Perkel and Bullock, 1968).

3 See Ryskin and Nieuwland (2023) for an expanded list of hypotheses for a PC model in language, although these were not meaningfully different from Walsh et al. (2020).

4 Omission responses will be discussed in Hypothesis 2.

5 For expected stimuli, there is a necessary correlation between sentence constraint and cloze probability. However, we can dissociate the effect of contextual constraint for unexpected targets because the context can be broadly unconstrained (i.e., weak or low constraint) or constrained toward a high cloze probability competitor (i.e., the difference between low-constraint unexpected and high-constraint unexpected). In these cases, cloze probability is matched, but only high-constraint unexpected is a prediction violation, and the N400 is not different across these contexts.

6 Some however, suggest amplitude modulations relate more to updates to the generative model (e.g., Bornkessel-Schlesewsky and Schlesewsky, 2019).

7 These article effects appear more robust in languages with gender-marked or case-marked adjectives (e.g., Spanish, Dutch, German).

8 Some of the following cited studies included only unexpected-anomalous target words, while others differentiated unexpected-plausible and unexpected-anomalous targets. The general pattern of N400 amplitude follows a gradation, such that expected < unexpected-plausible < unexpected-anomalous (or incongruent). For the remainder of the manuscript, we use unexpected to refer exclusively to the unexpected-plausible condition.

9 Semantic anomalies (of which orthographic neighbors are one specific type) are both unexpected (and therefore unpredictable) and implausible in a sentence context. Even though anomalies by definition are unpredictable, the mechanisms underlying *implausible* words and *plausible* prediction violations (also unexpected) are thought to be distinct. There's evidence of both behavioral (e.g., Rayner et al., 2011) and neural dissociation of these effects (e.g., DeLong et al., 2014a).

10 See (Kamaleddin 2026), an editorial on a Research Topic on multiplexed encoding.

11 This section could have also been placed under Hypothesis 2 (Prediction). We have chosen to include it under Hypothesis 4 (Hierarchical Inference) because of the dynamics across hierarchical levels that the frontal positivity is purported to elicit and because of the possible top-down precision modulation by task goals and experience.
